# The proposed Code of Conduct for Research in South Africa: despite good progress, unresolved issues remain

**DOI:** 10.1057/s41599-024-02715-0

**Published:** 2024-02-10

**Authors:** Amy Gooden, Donrich Thaldar

**Affiliations:** 1 School of Law, University of KwaZulu-Natal, Durban, South Africa.

## Abstract

After a 3-year development process and several drafts, the Academy of Science of South Africa (ASSAf) has submitted its proposed Code of Conduct for Research (proposed CCR) to the South African Information Regulator for its consideration and approval. When approved, the proposed CCR will be an important legal instrument that will complement the Protection of Personal Information Act 4 of 2013 (POPIA) in governing research activity in the country—including data sharing by South African researchers with their collaborators in other countries. The proposed CCR resolves important issues that were present in previous drafts. However, three important issues require attention: (1) how the identifiability of data subjects is to be determined in research data; (2) how research data can be repurposed for commercial use; and (3) how open access genomic databases should be established in the South African legal framework. In addition, the proposed CCR introduces a new issue: a legally unsustainable exception from POPIA application for genetic data. All these issues considered, the proposed CCR needs revision ahead of its approval by the Information Regulator. Recommendations are made on how to resolve the remaining issues.

## Introduction

In recent years, legislation relating to data protection has grown with the enactment of statutes, such as the European Union (EU) General Data Protection Regulation 2016/679 (GDPR), Canada’s Personal Information Protection and Electronic Documents Act of 2000 (PIPEDA), the California Consumer Privacy Act of 2018 (CCPA), and Brazil’s General Data Protection Law of 2018. In South Africa, the Protection of Personal Information Act 4 of 2013 (POPIA) has taken centre stage. POPIA is South Africa’s primary legal instrument dealing with the protection of, and access to, data (ASSAf, 2018). POPIA safeguards personal information and thereby privacy, which is a right recognised under section 14 of the Constitution of the Republic of South Africa, 1996 (the Constitution). POPIA aims to give effect to this right, while balancing it against the right of access to information contained in section 32 of the Constitution. POPIA places duties on those who request, collect, process, store, and use personal information. These parties (*responsible parties* in terms of POPIA) are bound not only by POPIA, but are also required to comply with any relevant codes of conduct issued in terms of Chapter 7—which serve to clarify and guide the interpretation of POPIA in a particular sector.

Recently, after several drafts, the Academy of Science of South Africa (ASSAf) submitted its proposed Code of Conduct for Research (the proposed CCR) in terms of POPIA to the Information Regulator for its consideration and approval. On 12 May 2023, the Information Regulator published the proposed CCR in the *Government Gazette* for public comment. The proposed CCR applies to all responsible parties who process personal information for the purposes of research (ASSAf, 2023), and seeks to, inter alia, help ensure legal certainty and compliance with the relevant provisions in POPIA, promote accountability for non-compliance, and safeguard research participants and research data in South Africa (ASSAf, 2023).

In this article, we begin by providing a background to POPIA. We then consider codes of conduct in terms of POPIA and their relevance. Following this, we consider the proposed CCR. We compare it with previous drafts, and assess it from both a legal and practical perspective. This is done by highlighting the salient issues that were remedied in the proposed CCR and note several issues that are insufficiently addressed. We also discuss a new issue apparent in the proposed CCR: a possible special exception for genetic data.

## The history of POPIA

Before proceeding with an analysis of the proposed CCR, we describe the background and the context within which POPIA emerged. Prior to POPIA, South Africa lacked specific data protection legislation. In 2009, the South African Law Reform Commission (2009) recommended that South Africa should enact data protection legislation, in line with international developments. Following this, the Protection of Personal Information Bill (2009) was tabled in the South African parliament. In 2013, parliament assented to POPIA—which was influenced by, inter alia, the Organisation for Economic Co-operation and Development (OECD) Guidelines on the Protection of Privacy and Transborder Flows of Personal Data (1980), and the Council of Europe Convention for the Protection of Individuals with regard to Automatic Processing of Personal Data (1981). Although enacted, POPIA entered into force in stages. This was to allow for the establishment of the Information Regulator (POPIA’s enforcement mechanism) and to give South Africans time to ensure POPIA compliance (Thaldar and Townsend, 2021). Most of POPIA’s provisions entered into force on 1 July 2020 (proclamation R21 of GG 43461, 2020), but given the one-year grace period in section 114(1) of POPIA, actual enforcement was from 1 July 2021.

POPIA aims to protect and regulate personal information processed by responsible parties, subject to justifiable limitations and conditions prescribing minimum thresholds for the lawful processing of personal information (section 2). It applies to all responsible parties in South Africa that process personal information and contains rights for individuals (known as *data subjects*) and duties for responsible parties. Responsible parties may only lawfully process personal information in line with the eight conditions in POPIA, these being accountability (section 8), processing limitation (sections 9 to 12), purpose specification (sections 13 and 14), further processing limitation (section 15), information quality (section 16), openness (sections 17 and 18), security safeguards (sections 19 to 22), and data subject participation (sections 23 to 25). Responsible parties must ensure that personal information is complete, precise, and updated where required (section 16). Personal information must be processed in a manner that is reasonable, not excessive, and in accordance with the given purpose (sections 9 to 12). Personal information must be stored and discarded securely, and data subjects must be informed about how their information is used and processed. Data subjects also have rights to access, correction, and deletion (sections 23 and 24).

Although POPIA provides greater protection to personal information and the individuals to which it relates, certain areas may require further guidance. This is where a code of conduct offers a solution.

## Codes of conduct

As mentioned above, Chapter 7 of POPIA deals with codes of conduct. Codes of conduct seek to apply a statute’s provisions to a specific sector, and to promote adherence. According to section 60(2) of POPIA, a code of conduct must:

“(a) incorporate all the conditions for the lawful processing of personal information or set out obligations that provide a functional equivalent of all the obligations set out in those conditions; and

(b) prescribe how the conditions for the lawful processing of personal information are to be applied, or are to be complied with, given the particular features of the sector or sectors of society in which the relevant responsible parties are operating.”

Codes of conduct aim to guide the interpretation of POPIA for a specific sector or industry (Adams et al. 2021). In 2020, ASSAf—as the official national Academy of Science of South Africa that is mandated by the Academy of Science of South Africa Act 67 of 2001, as amended by the Science and Technology Laws Amendment Act 16 of 2011, to offer scientific advice on matters of public interest to government and stakeholders and to use science to benefit society—began drafting a Code of Conduct for Research (CCR) in terms of POPIA. A first-draft CCR was published for public comment in 2021. However, it raised several concerns that were addressed by various academics. These included special personal information and children’s information (Townsend and Thaldar, 2019), the meaning of responsible party (Swales et al. 2022), the definition of public interest (Thaldar, 2022), and whether specific consent in POPIA is inclusive of broad consent (Thaldar and Townsend, 2020a; Thaldar and Townsend, 2020b; Swales, 2022).

Following this, a second-draft CCR was published by ASSAf in 2022. Although this second-draft CCR had corrected some of the shortcomings of the first draft, there were still areas of concern that were once again raised in the literature. These included the interpretation of certain core concepts in POPIA (Thaldar et al. 2023a), the repurposing of research data for commercial use (Townsend et al. 2023), and the possibility of open access databases (Thaldar et al. 2023b).

The proposed CCR that was submitted to the Information Regulator on 19 April 2023 is therefore the third public draft. When and if approved by the Information Regulator, the proposed CCR will become legally binding. In what follows, we analyse various aspects of the proposed CCR and establish whether previous concerns and suggestions have been considered and attended to.

## Analysis of the proposed CCR

In the following paragraphs, we provide an analysis of various pertinent aspects of the proposed CCR. We begin by highlighting several issues, present in previous drafts of the CCR, that were resolved in the proposed CCR—namely, that consent in terms of POPIA is to be specific and not broad; that special personal information is a subclass of personal information; that researchers are classified as responsible parties; that terminology used in the proposed CCR should be consistent with that which is used in POPIA; and that determinations of adequacy in cross-border transfers of data be conducted locally.

We then examine certain issues that were not adequately addressed in the proposed CCR—namely, whether context is relevant in determining the identifiability of a data subject; that the proposed CCR lacks a pathway for the repurposing of research data for commercial use; and that there is no guidance on how open access genomics databases should be constructed—in line with open science principles.

Following this, we consider a new issue in the proposed CCR: a potential special exception for genetic data—something which is neither practically or legally tenable in South Africa, nor is it in line with POPIA.

## Issues that were resolved in the proposed CCR

Previous drafts of the proposed CCR contained certain problematic interpretations of core concepts. These included consent, special personal information, responsible party, and de-identification. These were highlighted by Thaldar et al. (2023a). Each of these concepts is discussed below.

### Specific vs broad consent.

Section 1 of POPIA defines *consent* as “any voluntary, *specific* and informed expression of will in terms of which permission is given for the processing of personal information” (own emphasis). Although this indicates that consent in POPIA is to be specific, some authors believe that POPIA can be interpreted as allowing broad consent (Staunton et al. 2019). The initial draft of the CCR provided that consent can be broad (Adams et al. 2021). Also, in the previous draft of the CCR, ASSAf (2022) stated (in table 4 under paragraph 4.3.3.3.5) that “POPIA Consent for future use is allowed as long as the future uses of the Personal Information are not speculative, are described as fully as possible, and further use of the Personal Information is restricted.” However, Thaldar and Townsend (2020a) and Swales (2022) noted that POPIA’s provisions cannot mean broad consent. Thaldar et al. (2023a) contend that this is not in line with POPIA, as “specific” and “not speculative” are different. In addition, given the research exceptions in sections 15(3)(*e*) and 27(1)(*d*)) of POPIA, consent for future research may not be required in terms of POPIA (Thaldar et al. 2023a). The proposed CCR no longer contains reference to broad consent and provides that consent in terms of POPIA must be specific—”the consent must relate to a specifically defined study; simply obtaining consent to ‘conduct research’ will not be sufficient” (in table 4 under paragraph 4.3.2.2.3) (ASSAf, 2023).

### Special personal information as a category of ordinary personal information.

POPIA regulates personal information and *special* personal information. Special personal information is a subclas*s* of personal information, which triggers an extra layer of protection (in addition to the rules applicable to personal information). However, Thaldar et al. (2023a) find that the previous draft of the CCR failed to make this distinction. It stated (in paragraph 4.3.3.3.5) that “Any of the following legal justifications [referring to the grounds listed in section 11 of POPIA for the processing of personal information] must apply when the Research does NOT include Special Personal Information” (ASSAf, 2022). This therefore erroneously seems to exempt special personal information from complying with section 11 of POPIA. The proposed CCR has amended this (in paragraph 4.3.2.2.3). It removed the above sentence, and replaced it with “Any of the following legal justifications must apply to the processing of Personal Information: Special Personal Information is a subclass of Personal Information. The processing of Special Personal Information or the Personal Information of Children must also be authorised in terms of section 27 or section 35, respectively. This is an additional safeguard” (ASSAf, 2023). This clarifies that special personal information is a subclass of personal information, which must comply with additional requirements.

### Is a researcher a responsible party?

Section 1 of POPIA defines a *responsible party* as “a public or private body or any other person which, alone or in conjunction with others, determines the purpose of and means for processing personal information.” In terms of research, responsible parties are likely to include individual researchers and research institutions. However, Thaldar et al. (2023a) find that the previous draft of the CCR proposes that a responsible party excludes an individual researcher employed by a research institution (ASSAf, 2022). Thaldar et al. (2023a) recommend that references to individual researchers employed by research institutions not being included as responsible parties be amended. This was done in the proposed CCR, which now refers to responsible parties as including public or private bodies directing their employees, researchers undertaking their own research, organisations that make joint decisions about research, and researchers and organisations that make joint decisions (ASSAf, 2023). The proposed CCR has also changed previous references to “research institutions and independent researchers” to “researchers” (ASSAf, 2023).

### Avoiding terminological contagion.

POPIA uses the term *de-identification* when referring to the deletion of any information that identifies a data subject. However, the previous draft of the CCR referred to de-identification and anonymisation—and the latter term is not used in POPIA. Although de-identification and anonymisation are sometimes seen as synonymous, their meanings differ (Swales, 2021). Furthermore, although other jurisdictions may use such terms in their data protection legislation (such as de-identification in the United States Health Insurance Portability and Accountability Act of 1996 (HIPAA) and the United Kingdom’s Data Protection Act 2018 (DPA)), their definitions differ from the meaning of de-identification in POPIA. According to Thaldar et al. (2023a), the inclusion of anonymisation in the previous draft of the CCR was problematic. They recommend that foreign terms such as anonymisation be deleted from the proposed CCR, reference to foreign terms and documents be explained, and reference to foreign tests be removed. The proposed CCR has removed all reference to anonymisation, and only POPIA’s de-identification remains. Furthermore, references in the proposed CCR to foreign tests and standards have been deleted.

### Localising adequacy determinations.

Transborder flows of personal information are important in research as data may be sent abroad. However, this has created some uncertainty about what is allowed. Section 72(1) of POPIA provides that, inter alia, in order to transfer personal information to another country, the third party in that country must provide an adequate level of protection. The previous draft of the CCR recognised that the country must have laws that are equivalent to POPIA (ASSAf, 2022). It also stated (in paragraph 4.3.10.1.1) that it “considers countries in the European Union or a country that has received an adequacy decision from the European Commission, as equivalent to POPIA” (ASSAf, 2022). This was problematic because the previous draft of the CCR relied on the EU to make decisions about adequacy. The proposed CCR has now amended this (in paragraph 4.3.9.2) to state that “POPIA allows for the cross-border transfer of Personal Information if the recipient of the information is ‘subject to a law… which provide[s] an adequate level of protection.’ The proposed CCR recognises that researchers are not equipped to make such an adequacy determination. ASSAf will establish a committee to develop criteria for adequacy assessments in the research context” (ASSAf, 2023). It is positive that ASSAf has placed determinations of adequacy for POPIA in local hands instead of relying on the decisions of foreign jurisdictions.

## Issues insufficiently addressed in the proposed CCR

### The role of context in the determination of identifiability.

The most notable issue that remains unresolved is whether the identifiability of a data subject from information should be determined context specific (the data subject can be identified by a specific person) or context agnostic (the data subject can be identified by someone somewhere in the world). This issue is critical in research, as it will determine whether pseudonymisation is sufficient to make a dataset de-identified in the hands of the receiver of such a dataset, such as a research collaborator. For example, say researcher A pseudonymises a dataset—i.e., the researcher replaces all identifying information in the dataset with an alphanumeric code, and creates a second dataset (the identification key) that links the alphanumeric code with the identifying information. Researcher A shares the pseudonymised dataset with Researcher B, but keeps the identification key confidential. Is the pseudonymised dataset that Researcher B received personal information, or is it non-personal information? The answer will determine whether POPIA is applicable to the pseudonymised dataset in the possession of Researcher B.

- A context-agnostic approach—can the data subjects can be identified by someone somewhere in the world? Clearly, someone somewhere in the world, Researcher A, has the identification key and can therefore identify the data subjects in the pseudonymised dataset. Thus, POPIA applies to the pseudonymised dataset in the possession of Researcher B.
- A context-specific approach—can the data subjects be identified by the holder of the dataset? In the hands of Researcher B, the data subjects in the pseudonymised dataset cannot be identified. This means that POPIA does not apply to the pseudonymised dataset in the possession of Researcher B.

The unresolved question of whether context is relevant is not unique to South Africa. In Single Resolution Board v European Data Protection Supervisor (2023), for example, the General Court of the EU ruled against the European Data Protection Supervisor and adopted a context-specific approach to determining whether pseudonymised data were personal data in the hands of a receiver. However, the judgment has been appealed by the European Data Protection Supervisor (2023).

In the absence of case law in South Africa, how should POPIA be interpreted? Similar to the GDPR in the EU, POPIA does not explicitly state whether context is relevant in determining whether data subjects are identifiable from data. However, based on an analysis of POPIA, Thaldar (2023) suggests that the context-specific approach is the intended approach. Thaldar (2023) notes that POPIA’s research exception (in section 15(3) (*e*)) provides that researchers can publish their underlying data in de-identified form while, at the same time, retaining the underlying data in identifiable form. This means that POPIA contemplates a situation where the same data have a parallel existence as (a) non-personal information *in the hands of* the general public excluding the publishing researchers, and as (b) personal data *in the hands of* the publishing researchers. This means that the nature of data (personal data or non-personal data) is determined *in the hands of* the person holding the data—i.e., the context-specific approach. As Thaldar (2023) argues further, if POPIA contemplated a context-agnostic approach, the provision that researchers can publish their underlying data in *de-identified* form would only be possible if they also *de-identified* the data in their own hands. This is clearly not contemplated in POPIA’s research exception.

To create legal certainty in the research context, we suggest that the proposed CCR should adopt the context-specific approach and provide detailed examples of how it will function in research practice.

### Repurposing research data for commercial use.

Data collected for commercial purposes are commonly re-used for research. This is often the case in commercial biobanks. However, what legal pathway is there when research data are repurposed for commercial use? The commercialisation of research is an important public policy objective in South Africa, as evidenced by policies such as the Bio-economy Strategy (2013) and even statutes such as the Intellectual Property Rights from Publicly Financed Research and Development Act (2008).

The repurposing of research data for commercial use was addressed by Townsend et al. (2023) who examine this issue with reference to POPIA and the previous draft of the CCR, which lacked a pathway for repurposing research data for commercial use. They find that POPIA is both a foil and a facilitator for the commercialisation of research data. Although POPIA provides lists of instances where further processing is either compatible (section 15(2)) or not incompatible (section 15(3)) with the purpose of collection, the situation and whether commercialisation is allowable will need to be determined on a case-by-case basis. However, Townsend et al. (2023) recommend that, given the importance of the commercialisation of research to South Africa, the previous draft of the CCR should be amended to include situations where personal information initially collected for research is commercialised.

Despite this, the proposed CCR remains silent on the matter. Thus, there is a lacuna for the research community which the proposed CCR does not address. The proposed CCR (like the previous draft) recognises that where there is re-use of personal information that was initially collected for another purpose, a further processing assessment must be undertaken (ASSAf, 2022; ASSAf, 2023). However, the proposed CCR further states (under table 6) that this further processing assessment is used to “determine whether the secondary use of the Personal Information is justified” (ASSAf, 2023). Although not referring to the re-use of research data for commercial purposes, the further processing assessment may allow for such.

As POPIA allows for research data containing personal information to be repurposed for commercial use, and given that the proposed CCR seeks to provide guidance to researchers—some of whom may wish to repurpose research data—we suggest that the proposed CCR provide greater clarity by establishing a pathway for doing so.

### Open science and open access databases.

As research using personal information and special personal information (pertinently, genomics research) becomes more prevalent, population-level genomics databases have grown. Many countries collect genotype-phenotype data from individuals to be stored in biobanks and used for various purposes, such as research. This has the potential to better population health and lead to the realisation of precision medicine (Khoury and Holt, 2021; National Human Genome Research Institute, 2020; Pang, 2002; Roberts et al. 2021).

Although many databases require approval from a data access committee before access can be granted, more open access databases have been explored. In terms of POPIA, Thaldar et al. (2023b) have found that there is indeed a legal pathway (and an ethics pathway) allowing for the establishment of such an open access genomics database in South Africa (Thaldar et al. 2023b; Gooden and Thaldar, 2023). This would involve individuals openly sharing their genomic data, but ensuring that there are protections. These protections include requiring individuals to consent to the uploading of their genomic data by providing them with information on the risks to their privacy and their rights in terms of POPIA, as well as requiring individuals to undergo an objective assessment of their understanding of such risks to ensure that their consent is truly informed. The open access database must also ensure that data downloaders register on the website, verify the registration information, and declare that the data will be used for research.

However, the proposed CCR does not mention a pathway for open science and open access databases. Open science promotes the free sharing of scientific knowledge and is defined as “research and development that is collaborative, transparent and reproducible and whose outputs are publicly available” (DSI, 2022). South Africa is committed to the idea of open science, which has been endorsed by the Department of Science and Innovation (DSI) 2022 draft National Open Science Policy (DSI, 2022). This seeks to, inter alia, support a shift in research that supports open science principles; publish publicly funded research outputs; increase collaboration; make research outputs accessible for re-use; and foster the participation of society in science and innovation (DSI, 2022).

“Open access” refers to “a set of principles and a range of practices through which research outputs are distributed online, free of cost or other access barriers” (DSI, 2022). However, the proposed CCR takes a more qualified view of the meaning of open access. According to the proposed CCR (table 7), open access data repositories can be subject to restrictions such as paywalls, or data access committees (ASSAf, 2023). We suggest that the proposed CCR’s interpretation of the meaning of “open access” is problematic, as it means that restricted access databases would incorrectly and confusingly be deemed “open access.” We suggest that this should be rectified in the proposed CCR. It should be aligned with the draft National Open Science Policy (DSI, 2022).

Moreover, it would assist researchers if the proposed CCR could—based on the principles identified by Thaldar et al. (2023b) mentioned above—elaborate with practical guidelines on how (truly) open access genomics databases should be constructed. This will assist tremendously in translating the country’s policy commitment to open science into practice.

## A new issue: a special exception for genetic data?

The proposed CCR introduces (in paragraph 1.5 of Annexure A) a far-reaching change to previous drafts: genetic data do not qualify as personal information if the data are not linked with other information that can directly or indirectly identify a living individual. This would mean that genetic data on their own would fall outside POPIA’s scope and can therefore be shared publicly at the will of the data owner. (As suggested by Thaldar et al. (2022), the research institution that generated the genetic data is best positioned to claim ownership of such data.) In effect, this creates a special exception for genetic data to fall outside POPIA’s scope of application. However, is this special exception for genetic data legally tenable?

The idea that genetic data should be viewed as personal information only if the genetic data are linked with other identifying information relates back to a recommendation made in a report by Mitchell et al. (2020) on genomic data and the GDPR. However, the authors’ reasoning is based on the probability that genomic data will be identified, which they—with good reason—deem not reasonably likely. However, this reasoning is not applicable in the context of POPIA. POPIA’s test for de-identification is whether there is a reasonably foreseeable method of identifying the data subject. A reasonably foreseeable method can exist—even if not reasonably likely to be used. Accordingly, it would be a mistake to adopt the recommendation made by Mitchell et al. (2020) in South African law.

Even in the UK, the recommendation made by Mitchell et al. (2020) would have an uphill battle. The authors themselves highlighted that the Information Commissioner’s Office (n.d.) states that:

“in practice, genetic analysis which includes enough genetic markers to be unique to an individual is personal data and special category genetic data, even if you have removed other names or identifiers.”

Accordingly, the idea of a special exception for genetic data is not the consensus legal position. Moreover, the reasoning underlying it is not applicable in the South African context.

We further suggest that there are strong arguments against a special exception for genetic data. At a practical level, the idea that genetic data are only personal information if they are linked with other identifying information is tantamount to saying that a photograph of a person is only personal information if the individual’s name is written on the photograph. As with genetic data, a person’s visage is unique (except in the case of identical twins). However, if one is presented with an unknown data subject’s genetic data and photograph, one would be unable to identify the data subject from the genetic data or the photograph by just looking at it. Technical tools would be needed, and the success of these tools would depend on other information. If the data subject avoids having his/her picture published online, identifying him/her may be difficult if not impossible. Similarly, the chance of finding a genetic match in cyberspace may be slim, but not impossible. Thus, in the case of both the photograph and the genetic data there are certain practical factors that would influence the difficulty of identifying the data subject. Yet, in principle, the data subject can be identified by either the photograph or the genetic data. Thus, why is the proposed CCR making a special exception for genetic data?

We also suggest that a special exception for genetic data, as introduced in the proposed CCR, would be in conflict with POPIA. POPIA provides that personal information includes biometric information; biometrics, in turn, is defined as including DNA analysis. Since the output of DNA analysis is genetic data, clearly genetic data qualify as personal information. Therefore, the special exception for genetic data that the proposed CCR seeks to introduce is misaligned with POPIA. Importantly, a code of conduct cannot restrict the privacy rights that data subjects enjoy in terms of POPIA. Accordingly, the special exception for genetic data should be struck out by the Information Regulator before approving the proposed CCR. Even if hypothetically approved by the Information Regulator, the special exception for genetic data would not be legally valid, as it is in conflict with the primary legislation—POPIA. Such a scenario would harm genetics research, as researchers would believe that genetic data that they generate are not governed by POPIA, as long as the genetic data are not linked with other identifying information. Treating genetic data as non-personal information could expose genetics researchers and their institutions to litigation by data subjects.

## Conclusion

The proposed CCR has been through several drafts—and it has included amendments that have been both positive and negative. Problematic issues were highlighted in previous drafts of the CCR. These have now been amended in the proposed CCR: (1) consent in POPIA is to be interpreted as specific and not broad;(2) special personal information is a subclass of personal information and must comply with the requirements relating to both personal information and special personal information; (3) individual researchers are considered responsible parties; (4) references to terminology foreign to POPIA (specifically anonymisation) are deleted from the proposed CCR to avoid confusion; and (5) adequacy decisions about the cross-border transfer of data for research are to be made by a committee established by ASSAf, rather than relying on decisions of the EU.

However, certain issues are still problematic in the proposed CCR: (1) whether context is relevant to determining the identifiability of a data subject from information; (2) providing a pathway for repurposing research data for commercial use; and (3) open science and the establishment of open access databases. There is also the exception for genetic data as personal information in the proposed CCR, which is in conflict with POPIA. The proposed CCR is important and holds great potential to guide the research community in South Africa in terms of adherence to POPIA—especially if areas of concern are attended to before the final CCR is approved and published.

## Data Availability

Data sharing is not applicable to this article as no datasets were generated or analysed during the current study.
